# Rationale and design of the German-speaking myeloma multicenter group (GMMG) trial HD6: a randomized phase III trial on the effect of elotuzumab in VRD induction/consolidation and lenalidomide maintenance in patients with newly diagnosed myeloma

**DOI:** 10.1186/s12885-019-5600-x

**Published:** 2019-05-28

**Authors:** Hans Salwender, Uta Bertsch, Katja Weisel, Jan Duerig, Christina Kunz, Axel Benner, Igor W. Blau, Marc Steffen Raab, Jens Hillengass, Dirk Hose, Stefanie Huhn, Michael Hundemer, Mindaugas Andrulis, Anna Jauch, Andrea Seidel-Glaetzer, Hans-Walter Lindemann, Manfred Hensel, Stefan Fronhoffs, Uwe Martens, Timon Hansen, Mohammed Wattad, Ullrich Graeven, Markus Munder, Roland Fenk, Mathias Haenel, Christof Scheid, Hartmut Goldschmidt

**Affiliations:** 1 Asklepios Hospital Hamburg, Altona, Hematology, Oncology and Palliative Care, 22763 Hamburg, Germany; 2 University Hospital Heidelberg, Heidelberg, Germany; 3 National Center for Tumor Diseases Heidelberg, Heidelberg, Germany; 4 University Hospital Tuebingen, Tuebingen, Germany; 5 University Hospital Hamburg Eppendorf, Hamburg, Germany; 6 University Hospital Essen, Essen, Germany; 7 German Cancer Research Center Heidelberg, Heidelberg, Germany; 8 Charité Universitaetsmedizin Berlin, Berlin, Germany; 9 Institute of Pathology, Klinikum Ludwigshafen, Ludwigshafen am Rhein, Germany; 10 Institute of Human Genetics, University of Heidelberg, Heidelberg, Germany; 11 Cordination Center for Clinical Trials, University of Heidelberg (KKS), Heidelberg, Germany; 12 Kath. Krankenhaus Hagen, Hagen, Germany; 13 Mannheimer Onkologie Praxis, Mannheim, Germany; 14 Zentrum fuer ambulante Haematologie und Onkologie Siegburg, Siegburg, Germany; 15 SLK Klinikum Heilbronn, Heilbronn, Germany; 16 Ev. Krankenhaus Essen-Werden, Essen, Germany; 17 Krankenhaus Maria Hilf Moenchengladbach, Moenchengladbach, Germany; 18 University Medical Center Mainz, Mainz, Germany; 19 University Hospital Duesseldorf, Dusseldorf, Germany; 20 Klinikum Chemnitz, Chemnitz, Germany; 21 University Hospital Koeln, Cologne, Germany

**Keywords:** Multiple myeloma, elotuzumab, autologous stem cell transplant, high-dose chemotherapy

## Abstract

**Background:**

Despite major advances in therapy, multiple myeloma is still an incurable malignancy in the majority of patients. To increase survival, deeper remissions (i.e. CR) translating into longer PFS need to be achieved. Incorporation of new drugs (i.e. bortezomib and lenalidomide) as induction and maintenance treatment in an intensified treatment concept, including high dose melphalan (200 mg/m^2^), has resulted in increased CR rates, and is considered the standard of care for younger patients. Elotuzumab in combination with lenalidomide and dexamethasone has given better results as lenalidomide and dexamethasone alone in a phase III trial. The GMMG-HD6 trial will be the first phase III trial investigating the role of elotuzumab in combination with bortezomib, lenalidomide and dexamethasone (VRD) induction/consolidation and lenalidomide maintenance within a high dose concept.

**Methods:**

GMMG-HD6 is a randomized, open, multicenter phase III trial. The planned recruitment number is 564 NDMM patients. All patients will receive 4 VRD cycles as induction and undergo peripheral blood stem cell mobilization and harvesting. Thereafter they will be treated with high dose melphalan therapy plus autologous stem cell transplantation followed by 2 cycles of VRD consolidation and lenalidomide maintenance. Patients in arm B1 + B2 will additionally receive elotuzumab in the induction phase, whereas patients in A2 + B2 will be treated with elotuzumab added to consolidation and maintenance. The primary endpoint of the trial is PFS. Secondary objectives and endpoints are OS, CR rates after induction therapy comparing the two arms VRD (A1 + A2) vs VRD + elotuzumab (B1 + B2), CR rates after consolidation treatment, best response to treatment during the study, time to progression (TTP), duration of response (DOR), toxicity and quality of life.

**Results:**

Since this is the publication of a study protocol of an ongoing study, no results can be presented.

**Discussion:**

This phase III trial is designed to evaluate whether the addition of elotuzumab to an intensified treatment concept with high dose melphalan chemotherapy plus autologous stem cell transplantation and induction, consolidation and maintenance treatment with bortezomib and lenalidomide is able to improve PFS compared to the same concept without elotuzumab.

**Trial registration:**

NCT02495922 on June 24th, 2015.

## Background

Multiple myeloma (MM) is a malignancy of plasma cells and is the second most common hematological malignancy. The incidence rate in Europe is 4-7/100,000 per year. Approximately 5,700 cases are diagnosed in Germany each year [1]. As the disease progresses, morbidity and eventually mortality are caused by impaired immune system, skeletal destruction, anemia and renal failure. Despite major advances in therapy, multiple myeloma is still an incurable malignancy in the majority of patients.

Currently an intensified treatment concept including induction therapy, high dose melphalan (200 mg/m^2^) and subsequent consolidation/maintenance therapy is considered the standard treatment for MM patients up to the age of 70 years [1–4]. Incorporation of new drugs in induction treatment for tumor reduction and in maintenance therapy has resulted in increased CR rates. Median progression-free survival (PFS) after HDT in current standard approaches reaches 3 to 4, overall survival 9 to 11 years. Ten to 20% of intensively treated patients will be in remission more than 10 years [5]. Some of these long-term CR patients will likely be cured. To increase survival, deeper remissions (i.e. CR) translating into longer PFS need to be achieved, and patient outcome needs to be interpreted on the background of current substratification by molecular (gene expression profiling [GEP] and/or interphase fluorescence in situ hybridization [iFISH]) and imaging means.

The combination of bortezomib, lenalidomide and dexamethasone (VRD regimen) is a well-established three drug combination incorporating two “new drugs” for induction therapy in previously untreated multiple myeloma.

VRD has shown to be highly active and well tolerated [6–11] and is widely used in the US as standard therapy prior to and following HDT. VRD will be given in the GMMG-HD6 trial as background treatment for all study patients for induction therapy prior to a standard intensified therapy and for subsequent consolidation treatment.

Maintenance therapy after HDT prolongs duration of the response [12, 13]. The benefit of lenalidomide maintenance after HDT in terms of prolongation of PFS has been shown in several randomized trials [14, 15], with one of those also observing a superior OS [15]. Within the GMMG-HD6 trial, all patients are intended to receive lenalidomide maintenance.

Elotuzumab (BMS-901608; formerly known as HuLuc63) is a humanized recombinant monoclonal IgG1 antibody product directed to human SLAMF7 antigen (also known as CS1, CD2- subset-1), a cell surface glycoprotein that is highly expressed on MM cells. The proposed mechanism of action of elotuzumab involves natural killer (NK) antibody dependent cell-mediated cytotoxicity (ADCC), since elotuzumab kills MM cell lines in vitro in the presence of peripheral blood mononuclear cells (PBMCs) or purified NK cells. Because of its potent antitumor activity, elotuzumab is being developed for the treatment of MM. Data from a phase III trial (ELOQUENT-2) comparing elotuzumab in combination with lenalidomide and dexamethasone versus lenalidomide/dexamethasone alone, demonstrated an PFS advantage of 19.4 vs 14.9 months in relapsed/refractory MM patients [16, 17].

The combination of VRD plus elotuzumab is investigated by the Southwest Oncology Group in the US in a phase I/II trial for newly diagnosed high risk myeloma patients [18]. Another phase IIa study investigating the VRD plus elotuzumab combination in patients eligible for HDT and ASCT showed a low incidence of high grade toxicities, although there were two deaths in the study group [19]. Main research question of the GMMG-HD6 trial is to evaluate the effect of elotuzumab in induction /consolidation and maintenance therapy in previously untreated myeloma patients in a randomized setting. Given the previously described study results, an improvement of the therapeutic results by the addition of this humanized monoclonal antibody can be expected. Elotuzumab was the first antibody in myeloma with an FDA and EMA approval. To date, two phase III studies evaluate elotuzumab in first line and in 1^st^-3^*rd*^ relapse in the non-transplant setting. Results of phase I, II and phase III trials evaluating the combination of elotuzumab and bortezomib or lenalidomide and dexamethasone show very good tolerability and high response rates in patients with relapsed/refractory myeloma [20, 21]. While the results of use of elotuzumab in monotherapy were modest with stable disease as best response [22] the combination with lenalidomide and dexamethasone has given excellent results with *>*80% partial response in relapsed patients and prolonged PFS [20, 23, 24]. These data strongly support the evaluation of the combination of lenalidomide/dexamethasone plus bortezomib and elotuzumab within the context of an intensified therapy in newly diagnosed patients.

Within the GMMG-HD6 trial the best of four treatment strategies with respect to the PFS shall be determined. The four treatment strategies differ in the use of elotuzumab in addition to the background treatment of VRD induction/consolidation therapy (VRD +/- elotuzumab) and lenalidomide maintenance treatment (lenalidomide +/- elotuzumab), respectively.

The GMMG-HD6 trial will be the first phase III trial investigating the role of elotuzumab in combination with VRD and/or lenalidomide maintenance within a high dose concept. Results will be interpreted on the background of state of the art molecular profiling and imaging.

## Methods

### Design

GMMG-HD6 has a study population of 564 newly diagnosed multiple myeloma (NDMM) patients. It is a prospective, multicenter, randomized, parallel group, open, phase III clinical trial. There will be no blinding in this trial due to differences in the patient management in the treatment arms (premedication previous to application of elotuzumab and additional intravenous infusions for elotuzumab).

### Trial objectives

#### Primary objective

The primary objective of the study is the determination of the best of four treatment strategies regarding progression-free survival (PFS) - defined as time from randomization to progression or death from any cause whichever occurs first, censored at the end of the study.

The four treatment strategies are:

1. (arm A1): VRD (Bortezomib (Velcade) / Lenalidomide (Revlimid) / Dexamethasone) induction, intensification, VRD consolidation and lenalidomide maintenance,
2. (arm A2): VRD induction, intensification, VRD + elotuzumab consolidation and lenalidomide maintenance + elotuzumab,
3. (arm B1): VRD + elotuzumab induction, intensification, VRD consolidation and lenalidomide maintenance,
4. (arm B2): VRD + elotuzumab induction, intensification, VRD + elotuzumab consolidation and lenalidomide maintenance + elotuzumab.

#### Secondary objectives

The secondary objectives of this trial are to assess and to compare treatment arms regarding

- overall survival (OS)
- CR rates after induction therapy
- CR rates after consolidation treatment
- best response to treatment during the study
- MRD-negativity measured by flow (FACS) and next generation sequencing (NGS)
- time to progression (TTP), censored at end of trial
- duration of response (DOR), censored at end of trial
- toxicity during induction treatment, consolidation and maintenance treatment with respect to adverse events of CTCAE grade *≥* 3
- quality of life assessment of patients at baseline, during induction treatment, consolidation and maintenance treatment. Assessment of quality of life is performed using patient self-report questionnaires of the European Organization for Research and Treatment of Cancer Quality of Life Questionnaire (EORTC- QLQC30) including the multiple myeloma module (EORTC-QLQMY20).

### Setting

GMMG-HD6 is an investigator initiated trial by the German-speaking Myeloma Multicenter Group (GMMG) with a multicenter design.

### Estimated timeline

The duration of the trial for each patients is expected to be 36-39 months (induction and intensification treatment: 7-10 months, 3 months rest between intensification and start of consolidation, consolidation 2 months, maintenance phase 24 months) The overall duration of the trial is expected to be approximately 8 years including preparatory phase. Recruitment of patients has started in Q2 2015. The actual overall duration or recruitment may vary.

- Total trial duration: [96 months]
- Duration of the clinical phase: [74 months]
- Beginning of the preparatory phase: [Q1 2014]
- FPI (First Patient In): [Q4 2015]
- LPI (Last Patient In): [Q4 2017]
- LPO (Last Patient Out): [Q1 2021]
- DBL (Data Base Lock): [Q3 2021]
- Statistical analyses completed: [Q4 2021]
- Trial report completed: [Q1 2022]

### Ethical aspects, safety, consent

This study protocol is compliant with the declaration of Helsinki, the International Conference on Harmonization of Good Clinical Practice (ICH-GCP) guidelines, German law, regulations and organizations. A Data Safety Monitoring Board (DSMB) was installed before the start of the trial. The ethics committees/institutional review boards of all study sites gave a written approval before the start of this study.

Patients have to give written informed consent before any procedures regarding the trial are performed.

In the patient case report forms AEs are recorded and an assessment by the Investigator will be performed regarding intensity (according to CTCAE v4.0), seriousness and relatedness to the medication that is provided in the study.

For serious AEs (SAEs) there is an extra form that has to be filled out by the Investigator. This form has to be sent to the study administration within 24 hours after detection of the SAE. If a Suspected Unexpected Serious Adverse Reaction (SUSAR) occurs it will be reported to all investigators, ethics committees and the federal authorities.

### Selection of trial patients

Inclusion and exclusion criteria are listed in Table 1.

**Table 1:** Inclusion and exclusion criteria for the GMMG-HD6 trial

Inclusion criteria	
Patients meeting all of the following criteria will be considered for admission to the trial:	
Confirmed diagnosis of untreated multiple myeloma requiring systemic therapy (diagnostic criteria (IMWG updated criteria (2014). For some patients systemic therapy may be required though these diagnostic criteria are not fulfilled. In this case the GMMG study office has to be consulted prior to inclusion.	
Measurable disease, defined as any quantifiable monoclonal protein value, defined by at least one of the following three measurements	
Serum M-protein *≥*10 g/l (for IgA *≥* 5 g/l)	
Urine light-chain (M-protein) of *≥*200 mg/24 h	
Serum FLC assay: involved FLC level *≥* 10 mg/dl provided sFLC ratio is abnormal	
Age 18–70 years inclusive	
WHO performance status 0–3 (WHO = 3 is allowed only if caused by MM and not by co-morbid conditions)	
Negative pregnancy test at inclusion (women of childbearing potential)	
For all men and women of childbearing potential: patients must be willing and capable to use adequate contraception during the complete therapy. Patients must agree on the requirements regarding the lenalidomide pregnancy prevention programme.	
All patients must	
agree to abstain from donating blood while taking lenalidomide and for 28 days	
following discontinuation of lenalidomide therapy	
agree not to share study drug lenalidomide with another person and to return all	
unused study drug to the investigator or pharmacist	
Ability of patient to understand character and individual consequences of the clinical trial	
Written informed consent (must be available before enrollment in the trial)	
Exclusion criteria	
Patients presenting with any of the following criteria will not be included in the trial:	
Patient has known hypersensitivity to any drugs given in the protocol, notably bortezomib, lenalidomide, dexamethasone and elotuzumab or to any of the constituent compounds (incl. Boron and mannitol).	
Systemic AL amyloidosis (except for AL amyloidosis of the skin or the bone marrow)	
Previous chemotherapy or radiotherapy during the past 5 years except local radiotherapy in case of local myeloma progression. (Note: patients may have received a cumulative dose of up to 160 mg of dexamethasone or equivalent as emergency therapy within 4 weeks prior to study entry.)	
Severe cardiac dysfunction (NYHA classification III-IV, see appendix IIIB)	
Significant hepatic dysfunction (serum bilirubin *≥*1,8 mg/dl and/or ASAT and/or ALAT	
*≥* 2.5 times normal level), unless related to myeloma. (Note: if the mentioned limits for bilirubin or ASAT/ALAT are exceeded, but there is no significant hepatic dysfunction at investigator’s discretion, the GMMG study office has to be consulted prior to inclusion)	
Patients with renal insufficiency requiring hemodialysis	
HIV positivity	
Patients with active or history of hepatitis B or C	
Patients with active, uncontrolled infections	
Patients with peripheral neuropathy or neuropathic pain, CTC grade 2 or higher (as defined by the NCI Common Terminology Criteria for Adverse Events (NCI CTCAE) Version 4.0, see appendix V)	
Patients with a history of active malignancy during the past 5 years with the exception of basal cell carcinoma of the skin or stage 0 cervical carcinoma treated with curative intent	
Patients with acute diffuse infiltrative pulmonary and/or pericardial disease	
Autoimmune hemolytic anemia with positive Coombs test or immune thrombocytopenia	
Platelet count *<* 75 × 109/l, or, dependent on bone marrow infiltration by plasma cells, platelet count *<* 30 × 109/l (patients with platelet count *<* 75 × 109/l, but *>* 30 × 109/l may be eligible if percentage of plasma cells in bone marrow is *≥*50%), (transfusion support within 14 days before the test is not allowed)	
Haemoglobin *<* 8.0 g/dl, unless related to myeloma	
Absolute neutrophil count (ANC) *<* 1.0 × 109/l (the use of colony stimulating factors within 14 days before the test is not allowed), unless related to myeloma	
Pregnancy and lactation	
Participation in other clinical trials. This does not include long-term follow-up periods without active drug treatment of previous studies during the last 6 months.	
No patient will be allowed to enrol in this trial more than once.	

### Trial procedures

#### Randomisation and stratification

The patient has to be registered before the start of therapy. Patients need to be registered at the GMMG study office by sending the “Registration and Randomisation Form” by fax.

The following lab results are already necessary at registration, in addition to information regarding the eligibility criteria and the investigational site:

- Serum *β*-2 microglobulin value
- Serum albumin value
- Serum M-protein (concentration of monoclonal protein in serum)
- Urine M-protein (Bence Jones).

All eligibility criteria will be checked with a checklist. ISS stage will be calculated from the provided serum *β*-2 microglobulin value and serum albumin value in serum. If the patient needs to be registered before the requested lab results are available, the GMMG study office has to be consulted so that the patient can be included. The necessary laboratory investigations have to be initiated before start of treatment and the results have to be submitted to the GMMG study office as soon as possible. Each patient will be given a unique patient study number (“randomisation number”). Patients will be randomized using block randomization stratified by ISS stage (I vs. II vs. III) in order to achieve a balance of treatment groups with respect to this prognostic covariate. There will be no additional stratification by center. Influence of this covariate is considered to be less because of the long trial experience of most centers within the GMMG.

The probability for assignment in each of the four treatment arms (A1, A2, B1, B2) is 25%, the relation of treatment arms is 1:1:1:1.

Patient study number and result of randomization will be sent to the investigator by fax.

#### Screening

All patients have to undergo physical examination including assessment of WHO performance score, body weight, body height and concomitant diseases.

Before the inclusion into the trial for a patient is possible, the following laboratory investigations are necessary: C reactive protein, lactate dehydrogenase, *β*-2 microglobulin, albumin, total protein, pregnancy test (only for woman in childbearing age), immunoglobulins, monoclonal protein and free light chains in serum, monoclonal protein in urine, immunofixation in serum, immunofixation in urine, complete blood count including Absolute Neutrophil Count (ANC), electrolytes, renal parameters, hepatic parameters, thyroid stimulating hormone.

A bone marrow puncture has to be performed for bone marrow aspiration (cytology, iFISH) and bone marrow histology.

For the documentation of the skeletal status medical imaging with low dose, whole body computed tomography or conventional X-ray imaging is required.

An ECG and an echocardiogram have to be performed prior to study inclusion to document the cardiac condition of the patient.

#### Study visits

Monitoring will be done by personal visits from a clinical monitor according to SOPs of the coordination centers for clinical trials (KKS).

The monitor will review the entries into the CRFs on the basis of source documents (source data verification). The investigator must allow the monitor to verify all essential documents and must provide support at all times to the monitor.

By frequent communications (letters, telephone, fax), the site monitor will ensure that the trial is conducted according to the protocol and regulatory requirements.

#### Timepoints of clinical evaluations

The Table 2 shows the recommended timepoints of response evaluation. A non-essential deviation of these timepoints (e.g. for logistic reasons) is accepted, but it is important that the response to a treatment period will be assessed previous to the start of the subsequent period.

**Table 2:** Recommended timepoints of response evaluation

Treatment period	Timepoint of response evaluation
After induction treatment	d21 – d35 after start of 4*th* cycle VRD
After first chemotherapy cycle of intensification regime (mobilization)	d23 – d33 after start of this cycle (previous to high dose therapy)
After subsequent chemotherapy cycles of inten sification regime	According to local policy: d60 - d90 after start of high dose therapy cycle recommended (previous to next cycle)
After VRD consolidation	d21–35 after start of 2*nd* cycle VRD
During maintenance	every 3 months
Follow up, i.e. after individual “end of study”, (until 1*st* PD)	Evaluation is recommended every 3 months. As long as there is no change in response status, es- pecially no progressive disease, CRF documentation of the FU visit is sufficient every 6 months. The timepoint of diagnosis of progressive disease has to be documented in the eCRF, even if it doesn’t correspond to a regular 3- or 6-month visit, respectively.
Follow up, after 1st PD	There are no specific recommendations for the evaluation schedule after 1st PD as course of the disease and therapies for relapsed and/or refractory myeloma vary and the visits have to be adapted to the requirements of the individual patient. The requirements for eCRF documentation in FU after 1st PD are as follows: update of the survival status at least every 6 months (incl. Information on secondary primary malignancies and on the timepoint of diagnosis of subsequent “PD”). Each line of therapy should be recorded separately

#### Trial treatment

**Fig. 1:**
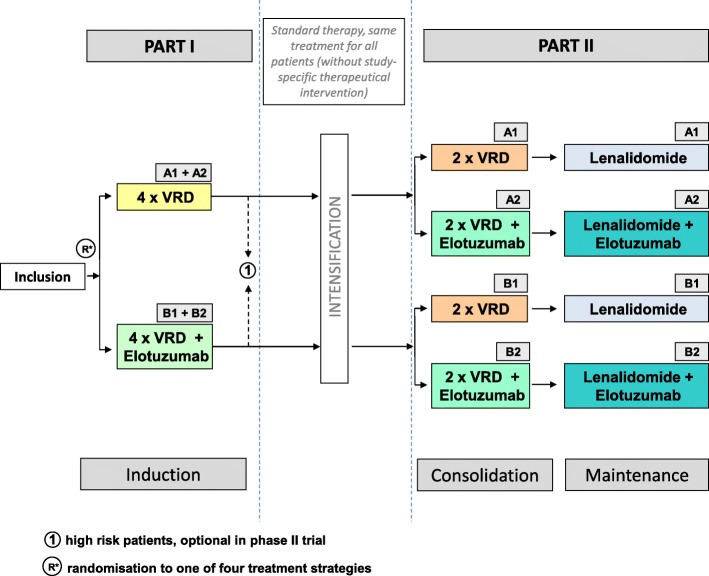
GMMG HD6 trial overview

Figure 1 includes a compact overview of the trial treatment.

After the inclusion in the study, all patients regardless of randomization will receive an induction treatment consisting of 4 VRD cycles of 21 days each (bortezomib 1.3 mg/m^2^ s.c. on days 1,4,8,11, oral lenalidomide 25 mg on days 1-14, oral dexamethasone 20 mg on days 1,2,4,5,8,9,11,12, additionally in cycle 1 and 2 on day 15). Patients in arm B1 and B2 will additionally be treated with elotuzumab 10 mg/kg on days 1,8,15 in cycle 1 and 2, days 1 and 11 in cycles 3 and 4.

All eligible patients will be given an intensified therapy regime according to GMMG standard protocols. A commonly used regimen for intensified therapy is: stem cell collection after CAD mobilization followed by high dose melphalan (200 mg/m^2^) and autologous stem cell rescue. GMMG standard is a single HDT and ASCT for patients who reach at least a CR and tandem HDT and ASCT for patients who do not reach CR. Although the details of stem cell collection, HDT and ASCT are not specified in the study protocol.

Three months after the beginning of the last HDT cycle patients should receive consolidation treatment consisting of 2 cycles of VRD of 21 days each with weekly administration of bortezomib (bortezomib 1.3 mg/m^2^ s.c. on days 1,8,15, oral lenalidomide 25 mg on days 1-14, oral dexamethasone 20 mg on days 1,2,8,9,15,16). Patients in arm A2 and B2 will be treated with elotuzumab 10 mg/kg additionally in both cycles on days 1,8,15.

Maintenance therapy should start on d35 of the second VRD consolidation cycle and will be given until confirmed progression, for 2 years or until unacceptable toxicity, whichever occurs first. All patients should receive 26 cycles lenalidomide maintenance every 28 days (oral lenalidomide 10 mg d 1-28) together with dexamethasone (12 mg on days 1 and 15 in cycle 1-6 and day 1 thereafter). Patients in arm A2 and B2 will be treated with Elotuzumab 10 mg/kg i.v. additionally on days 1 and 15 in cycle 1-6 and day 1 thereafter). A two years maintenance therapy timeframe was chosen to define a clear endpoint for the trial as required by the German authorities. Benefits of prolonged maintenance therapy beyond 2 years had not been shown in trials at the time of submission of this study protocol. Nonetheless, after the end of the study, all patients not encountering disease progression or inacceptable toxicity are suggested continuing lenalidomide maintenance treatment.

#### Supportive treatment

It is mandatory to give anti-viral (Aciclovir 2 x 400 mg/d p.o.) and anti-bacterial (Cotrimoxazol 2 x 960 mg/d p.o. or Ciprofloxacin 2 x 500 mg/d p.o.) prophylactic treatment to all patients during induction and consolidation therapy. A venous thromboembolism (VTE) prophylaxis in conjunction with VRD (+/- elotuzumab) has to be given. It is recommended to give acetylsalicylic acid (ASA) 100 mg daily. Patients at an individual high risk of thromboembolic events, such as patients with previous history of thromboembolism, should receive a VTE prophylaxis with low molecular weight heparin (LMWH). It is strongly recommended to start treatment with i.v. bisphosphonates at diagnosis and to continue this treatment every 4 weeks for at least 2 years. A commonly used regimen consists of zoledronate 4 mg or pamidronate 90mg once every 4 weeks. Elotuzumab requires premedication with H1 blocker, H2 blocker and paracetamol administered 30-90 minutes prior to elotuzumab administration and i.v. dexamethasone at least 45 minutes prior to elotuzumab.

#### Concomitant medication and treatment

Bisphosphonate treatment is recommended for all patients in the trial. It is permitted to treat trial participants with red blood- and platelet-transfusions, G-CSF and intravenous immunoglobulins. Furthermore, it is allowed to treat myeloma- or treatment-related complications (e.g. vertebroplasty in case of vertebral compression fracture). Radiotherapy is permitted. Additional use of substances with antineoplastic features is not allowed.

#### Follow up

Regular follow ups after the discontinuation of the study treatment are part of the study protocol. During this timeframe additional data regarding survival, toxicities, efficacy and subsequent myeloma-specific treatment will be gathered.

### Response assessment

Response will be assessed according to the International Myeloma Working Group (IMWG) uniform response criteria [25]. In addition and modification to the IMWG criteria “minimal response” (MR) as defined in the EBMT criteria [26], “near CR” (nCR) and “molecular CR” (mCR) have been added. According to the trial protocol, in case of a suspected CR based on routine testing at any time during therapy, a bone marrow puncture is performed to confirm the response. At the same time, MRD-analysis is performed. If CR is confirmed MRD assessment is repeated once after 6 months.

### Discontinuation criteria

#### Discontinuation of trial participation of individual patients

The patient can discontinue the trial treatment at any point without having to give any reason. The investigator can stop the trial treatment for patients if further treatment could be harmful or disadvantageous for the patient, if exclusion criteria are met, if a SAE occurs that precludes further treatment, if a trial participant gets pregnant, if data acquisition is not possible due to patient incompliance and if serological PD or PD with end organ damage (CRAB criteria [27]) is present in the trial patient. The only exception for PD is the occurrence of PD after induction therapy or stem cell apheresis. In this case further treatment in the trial is possible.

#### Closure of individual trial sites

Individual trial sites can be closed if the data quality is not sufficient or if the trial site provides inadequate patient recruiting.

#### Premature termination of the trial

If unknown risks occur during the trial; or due to inadequate recruiting not enough patients are acquired to continue the study, the DSMB or the principal investigator may terminate the trial prematurely.

### Statistical analysis

#### Sample size calculation

The GMMG-HD6 trial is designed to determine as primary objective the best of the four treatment arms with regard to progression-free survival (PFS), censored at end of trial. Assuming 2 years of recruitment, 3 years minimal follow-up time after end of recruitment, a total of 10% drop-outs and 5% high risk patients leaving the study prematurely after induction therapy, inclusion of 564 patients allows for rejecting the global null hypothesis of no difference between the four arms at the two-sided significance level of 5% with a power of 91%, if the arms achieve PFS rates of 60%, 70%, 70% and 80% after 3 years. This corresponds to hazard ratios relative to the worst arm of 0.698, 0.698 and 0.437. The PFS rates of the intermediate arms are conservatively chosen representing the least favorable distribution with respect to power. Further treatment comparisons will be realized within a closed testing procedure. The sample size calculation is based on the method of Barthel et al. for multi-arm survival trials [28]. An interim analysis with respect to PFS rates will be conducted after 2.5 years to rule out lack of efficacy. The strongest observed effect between the best and the worst arm will be used to recommend a potential stop for futility based on the conditional power (CP) as proposed by Lachin (2005) [29]. The study is recommended to be stopped if CP *≤* 20%. This possibility to stop for futility results in a power loss of maximally 9.6%, thus, the overall power to reject the global null hypothesis of no difference between the four arms achieves at least 81.4%. Assuming arm A1 as reference, we are mainly interested in the best-versus-reference comparison. Liu and Dahlberg [30] found for the comparison of the best versus reference arm that the power lies only slightly below the overall power of the study. Our own simulation studies under the specific study assumptions confirmed these results and showed 88.6% power for the best-versus-reference comparison. With the possibility to stop at interim if CP *≤* 20%, the power for the best-versus-reference comparison would still be larger than 79%.

#### Statistical methods

A detailed statistical analysis plan (SAP) will be finalized before closure of the database and the final analysis which has to be authorized by the biometrician, the sponsor, and the LKP. The analysis of the primary endpoint is confirmatory. All remaining analyses are exploratory and will be carried out at a two-sided significance level of 0.05 unless noted otherwise.

#### Primary endpoint

The primary endpoint is PFS, censored at end of trial. The four treatment arms will be compared in a closed testing procedure as introduced by Marcus, Peritz and Gabriel [26]. This hierarchical step-down approach controls the family-wise error rate in a multi-comparison setting if all null hypotheses are tested in a pre-defined hierarchical order at a the same significance level starting with the global null hypothesis of no treatment difference between the four arms down to the pairwise (elementary) null hypotheses of no treatment difference between two treatment arms. All null hypotheses will be tested confirmatory at the two-sided 5% significance level using the log-rank test stratified by ISS stage. Statistically significant different PFS of a treatment arm with respect to a comparator arm will be concluded if the adjusted p-value of the elementary hypothesis is below 0.05.

The analysis of the primary objective is performed after database closure and will be presented in the final biometrical report.

## Discussion

An intensified treatment concept including induction therapy, high dose melphalan (200 mg/m^2^) and subsequent consolidation/maintenance therapy is considered the standard treatment for fit MM patients [1, 2].

Results of randomized phase III trials comparing HDT with treatment including new drugs showed a benefit for HDT in response, minimal residual disease (MRD) and PFS [10, 31–33]. The data from the French study showed a strong correlation between MRD negativity (NGS [Sequenta] and FACS) and the normalization of lesions in PET-CT before maintenance [34, 35]. Molecular data before treatment and MRD-monitoring during treatment will allow to define a subgroup of MM patients that can be cured. In addition to testing the anti-SLAMF7 antibody elotuzumab in upfront induction and maintenance treatment of transplant-eligible patients, the CD38 targeting antibodies daratumumab and isatuximab are currently tested in ongoing trials, e.g. by the Intergroupe Francophone du Myélome (IFM) (Cassiopeia, NCT02541383).

Given the previously described study results in the relapsed setting, an improvement of the therapeutic results by the addition of the humanized monoclonal antibody elotuzumab can be expected. This phase III trial is designed to evaluate whether the addition of elotuzumab to an intensified treatment concept with high dose melphalan chemotherapy plus autologous stem cell transplantation and induction, consolidation and maintenance treatment with bortezomib and lenalidomide is able to improve PFS compared to the same concept without elotuzumab.

## Conclusion

We present the study protocol of the first phase III investigator initiated trial to explore the role of elotuzumab in combination with VRD and lenalidomide maintenance together with high dose melphalan and stem cell transplantation.

Results will be analyzed in comparison with molecular profiling and imaging.

## Abbreviations

ADCC: Antibody dependent cell-mediated cytotoxicity
AE: Adverse event
ANC: Absolute neutrophil count
ASA: Acetyl salicylic acid
ASCT: Autologous stem cell transplantation
CP: Conditional power
CR: Complete response
CRAB: Calcium elevation/renal failure/anemia/bone lesions
CT: Computed tomography
CTCAE: Common Terminology Criteria of Adverse Events
DOR: Duration of response
DSMB: Data safety monitoring board
ECG: Electrocardiogram
Elo: Elotuzumab
FPI: First patient in
GEP: Gene expression
GMMG: German-speaking Myeloma Multicenter Group
HD(C)T: High dose (chemo)therapy
ICH-GCP: International Conference on Harmonization of good clinical practice
iFISH: Interphase fluorescence in situ hybridization
IMWG: International Myeloma Working Group
ISS: International staging system
ITT: Intent-to-treat
KKS: Coordination Center for Clinical Trials (Koordinierungszentrum für Klinische Studien)
LKP: Coordinating investigator (Leiter der Klinischen Prüfung)
LMWH: Low molecular weight heparin
LPI: Last patient in
LPO: Last patient out
MM: Multiple myeloma
MR: Minimal response
nCR: Near CR
NDMM: Newly diagnosed Multiple Myeloma
NK: Natural killer
ORR: Overall response rate
OS: Overall survival
PBMC: Peripheral blood mononuclear cells
PD: Progressive disease
PFS: Progression free survival
RR: Response rate
SAE: Serious adverse event
SAP: Statistical analysis plan
SLAMF7: SLAMF7 gene
SOP: Standard operation procedure
SUSAR: Suspected unexpected serious adverse reactions
TTP: Time to progression
VGPR: Very good partial response
VRD: Bortezomib, lenalidomide and dexamethasone
VTE: Venous thromboembolism
WHO: World health organization
